# One–Two PM Punch: Gene Combination Increases Vulnerability to Air Pollutant

**DOI:** 10.1289/ehp.115-a551b

**Published:** 2007-11

**Authors:** Bob Weinhold

Numerous epidemiologic studies have found a link between exposure to fine particles (PM_2.5_) and increased morbidity and mortality. Other studies have found evidence of some of the specific pathways through which this damage occurs, but much remains unknown about the relative importance of these pathways in humans. An international team of researchers has taken a step toward filling that void through its discovery that the combination of two genetic traits—a deletion of one gene and a polymorphism of another—combine to significantly increase risk of oxidative stress and subsequent cardiovascular disease in some people exposed to PM_2.5_ **[*EHP* 115:1617–1622; Chahine et al.]**.

One well-known indicator of potential cardiovascular problems is reduced heart rate variability (HRV). When the heart is less able to vary its beat, it can’t respond as nimbly to challenges such as pollutants, microbes, or emotional stresses. PM_2.5_ exposure has been linked with reduced HRV, possibly in part by triggering oxidative stress.

Several genes are known to play a role in defending against oxidative stress. Two of these include glutathione *S*-transferase-M1 (*GSTM1*) and heme oxygenase-1 (*HMOX-1*). To determine whether these genes have a link with reduced HRV, the team studied 476 older Boston-area males, almost all white, for whom they had information on the two genes, as well as data for three established indicators of HRV: standard deviation of normal-to-normal intervals (SDNN), variation at high frequency (HF), and variation at low frequency (LF). Ambient PM_2.5_ data came from a central stationary monitor, which had sampled in the 48-hour period prior to HRV testing and had earlier been shown to be a good proxy for personal exposures in the area. The team accounted for confounders such as age, body mass index, smoking, and prescription drug use.

Overall, they found that each 10-μg/m^3^ increase in PM_2.5_ was associated with a statistically significant decrease in two measures of HRV (6.8% for SDNN and 17.3% for HF). Decreases for all three indicators were greater in men with either a *GSTM1* deletion or a particular polymorphism of *HMOX-1*. But the decreases were greatest in the men who had both the *GSTM1* deletion and the *HMOX-1* polymorphism, dropping 12.7% for SDNN, 27.8% for HF, and 20.1% for LF. This combination occurred in 48% of the subjects.

The researchers acknowledge that much more work needs to be done addressing variables such as sex, age, race, geographic setting, pathway of damage, and affected body system. But their findings lead them to conclude that at least one combination of genetic traits increases vulnerability to oxidative stress and cardiovascular damage from PM_2.5_.

